# Acid-Base and Photocatalytic Properties of the CeO_2_-Ag Nanocomposites

**DOI:** 10.3390/mi14030694

**Published:** 2023-03-21

**Authors:** Alexander A. Kravtsov, Andrey V. Blinov, Andrey A. Nagdalian, Alexey A. Gvozdenko, Alexey B. Golik, Maxim A. Pirogov, Maxim A. Kolodkin, Naiyf S. Alharbi, Shine Kadaikunnan, Muthu Thiruvengadam, Mohammad Ali Shariati

**Affiliations:** 1 Faculty of Physics and Technology, North Caucasus Federal University, 1 Pushkin Str., 355017 Stavropol, Russia; 2 Laboratory of Food and Industrial Biotechnology, Faculty of Food Engineering and Biotechnology, North Caucasus Federal University, 1 Pushkin Str., 355017 Stavropol, Russia; 3 Department of Botany and Microbiology, College of Science, King Saud University, Riyadh 11451, Saudi Arabia; 4 Department of Applied Bioscience, College of Life and Environmental Sciences, Konkuk University, Seoul 05029, Republic of Korea; 5 Department of Microbiology, Saveetha Dental College and Hospitals, Saveetha Institute of Medical and Technical Sciences, Saveetha University, Chennai 600077, Tamil Nadu, India; 6 Semey Branch of the Institute, Kazakh Research Institute of Processing and Food Industry, 238G Gagarin Ave., Almaty 050060, Kazakhstan

**Keywords:** CeO_2_, Ag, nanoparticles, nanocomposite, sol-gel, acid-base properties, photocatalytic properties

## Abstract

In this work, CeO_2_ nanoparticles, as well as CeO_2_ nanocomposites with plasmonic silver nanoparticles, were synthesized using a simple sol-gel process. The concentration of silver in the composites varied from 0.031–0.25 wt%. Cerium hydroxide dried gel was calcined at temperatures from 125 to 800 °C to obtain CeO_2_. It was shown that, at an annealing temperature of 650 °C, single-phase CeO_2_ nanopowders with an average particle size in the range of 10–20 nm can be obtained. The study of acid-base properties showed that with an increase in the calcination temperature from 500 to 650 °C, the concentration of active centers with pKa 9.4 and 6.4 sharply increases. An analysis of the acid-base properties of CeO_2_-Ag nanocomposites showed that with an increase in the silver concentration, the concentration of centers with pKa 4.1 decreases, and the number of active centers with pKa 7.4 increases. In a model experiment on dye photodegradation, it was shown that the resulting CeO_2_ and CeO_2_-Ag nanopowders have photocatalytic activity. CeO_2_-Ag nanocomposites, regardless of the silver concentration, demonstrated better photocatalytic activity than pure nanosized CeO_2_.

## 1. Introduction

Cerium oxide (CeO_2_) has gained widespread attention due to its many unique properties, including high chemical stability, excellent mechanical properties, thermal stability, and high oxygen conductivity. Its low cost also makes CeO_2_ an essential material for many applications in wide fields of science and technology [1]. Recently, nanostructured CeO_2_ has garnered the greatest attention due to its interesting effects in the nanoscale state.

CeO_2_ and nanostructured composites based on it have been widely studied as promising active materials for use in supercapacitors [2]. Additionally, CeO_2_ nanoparticles have been added to materials to improve their sensory properties, such as in the CeO_2_-MoS_2_ nanocomposite [3]. Combinations of nanomaterials based on CeO_2_ can also serve as effective adsorbents [4]. Studies have considered CeO_2_ as a modifying additive for different functional materials [5]. Besides, nanosized cerium oxide can be used as an activator for the production of phosphors, for example, YAG:Ce [6,7].

Nanosized CeO_2_ has great prospects for application in catalysis. CeO_2_ is an effective catalyst for oxidation and combustion processes due to its high rate of oxygen transfer. There are numerous studies devoted to the use of CeO_2_ in catalytic combustion processes. In some studies, CeO_2_ nanoparticles were used as an additive to biodiesel fuel to increase the completeness of fuel combustion and reduce harmful combustion products [8,9]. In another study, a hybrid catalyst was created based on spherical NiCuO_x_ bimetallic oxide nanoparticles decorated with a cluster of cerium nanoparticles (NiCuO_x_@CeO_2_) [10]. This catalyst showed excellent results in the catalytic combustion of methane.

Nevertheless, doping of oxide catalytic materials with plasmonic nanoparticles is known to increase the catalytic properties. Recent work has shown that Ag is an excellent dopant of TiO_2_ photocatalyst due to its striking advantages when compared to other noble metals. Studies have concluded that Ag-doped TiO_2_ successfully results in excellent charge transfer and the synergistic effect between SPR and metal oxides or heterojunctions enhances the resulting materials’ visible light responsiveness [11]. In another recent work, the addition of silver nanoparticles to zinc oxide made it possible to obtain a material with high photocatalytic properties. The authors indicate that Ag-decorated ZnO (ZnO/Ag) nano-photocatalysts are highly efficient and cost-effective in degrading organic pollutants [12]. Additionally, Pt/CeO_2_ catalysts based on nanosized CeO_2_ have been developed, which exhibit excellent activity and unique selectivity in various catalytic reactions due to their small particle size, a large number of unsaturated active sites, and unique electronic structures [13]. A recent work [14] produced a composite material by combining CeO_2_ nanorods with gold nanoparticles, which demonstrated remarkable catalytic activity in converting CO to CO_2_.

A comprehensive analysis of the literature revealed that CeO_2_-Ag nanocomposites have been extensively studied. Notably, CeO_2_ nanocomposites doped with plasmonic Ag, Au, and Pt nanoparticles have demonstrated promising photocatalytic properties [15]. Therefore, CeO_2_ nanocomposites decorated with plasmonic nanoparticles, especially silver nanoparticles, are highly promising materials with significant potential for application in various fields. However, the underlying mechanisms of CeO_2_ nanoparticles and their composites in various processes remain inadequately understood. Notably, no reference was found in the study of CeO_2_-Ag nanocomposites’ acid-base properties, which could provide critical information on the concentration of active sites of different natures. Such information is essential for understanding the catalytic, adsorption, and antimicrobial activity of these materials. Therefore, investigating the acid-base properties of CeO_2_-Ag nanocomposites is crucial for enhancing our understanding of these materials and improving their performance in various applications.

This study employed a straightforward sol-gel method to synthesize nanosized CeO_2_ particles with sizes ranging from 10 to 20 nm. We investigated the effect of calcination temperature on the acid-base properties of the nanosized CeO_2_, as well as the impact of silver concentration on the acid-base properties of the CeO_2_-Ag nanocomposite. Additionally, we analyzed the correlation between the acid-base properties and photocatalytic performance of CeO_2_-Ag nanocomposites.

## 2. Materials and Methods

### 2.1. Synthesis of CeO_2_ Nanoparticles

CeO_2_ nanoparticles were synthesized by the sol-gel method. The following procedure was used to prepare cerium oxide. Firstly, a solution of cerium nitrate was prepared, for which 126.15 g of cerium nitrate hexahydrate was dissolved in 400 mL of deionized water. An ammonia solution containing 174 mL of a concentrated 25% aqueous ammonia solution and 426 mL of deionized water was then prepared. For the synthesis of cerium hydroxide, a solution of cerium nitrate was added to the ammonia solution using a peristaltic pump at a rate of 7 mL/min with continuous stirring conditions. After the addition of the cerium nitrate solution, the mother liquor was further stirred for one hour to complete all chemical reactions and form a stable cerium hydroxide gel. Next, the gel was washed by centrifugation to a neutral pH value and dried in an oven at a temperature of 60 °C. Dry cerium hydroxide was calcined at temperatures of 125, 250, 500, 800 °C to obtain nanosized cerium oxide.

### 2.2. Synthesis of Ag Nanoparticles

A stable sol of silver nanoparticles was synthesized according to the author’s method described in earlier work [16]. Two solutions were prepared for the synthesis: the first solution was 0.1 g of silver nitrate in 15 mL of isopropyl alcohol, and the second solution was 0.05 g of sodium borohydride in 15 mL of isopropanol. The silver nitrate solution was stirred with a magnetic stirrer until the salt was completely dissolved. Then, 1 g of polyvinylpyrrolidone was added to the AgNO_3_ solution. The solution gradually turned orange-brown, indicating the onset of the reduction of silver under the action of PVP. After the complete dissolution of PVP, 2 mL of NaBH_4_ solution was slowly added dropwise to AgNO_3_ solution with vigorous stirring. In this case, a concentrated sol of silver nanoparticles of a saturated dark red color was formed.

### 2.3. Synthesis of CeO_2_-Ag Nanocomposite

Pure CeO_2_ powders were prepared by annealing dry Ce(OH)_4_ in a muffle furnace at temperatures of 125 °C, 500 °C, 650 °C, 800 °C. The CeO_2_-Ag composite was prepared as follows. Different amounts of silver nanoparticle sol were added to fresh Ce(OH)_4_ gel washed by centrifugation. A total of four samples were obtained with different concentrations of silver: CeO_2_-Ag1 (0.031 wt%), CeO_2_-Ag2 (0.064 wt%), CeO_2_-Ag3 (0.126 wt%), CeO_2_-Ag4 (0.25 wt%) and a control sample-pure CeO_2_ without silver. Then, the Ce(OH)_4_ gel with silver nanoparticles was thoroughly mixed for 2 h until complete homogeneity and then dried in an oven at a temperature of 60 °C. Dry Ce(OH)_4_-Ag powders were calcined in a muffle furnace at a temperature of 650 °C.

### 2.4. Research Methods

The morphology and elemental composition of the samples were studied using a MIRA 3 LMH (Tescan, Brno, Czech Republic) scanning electron microscope (SEM) with an Aztec Energy Standard/X-max 20 (standard) system for determining the elemental composition by energy-dispersive X-ray spectroscopy (EDX).The average hydrodynamic radius of silver nanoparticles was measured by photon-correlation spectroscopy (PCS) using a Photocor complex device (Photocor, Moscow, Russia).The phase composition of the samples was investigated by X-ray diffraction analysis on an Empyrean series 2 X-ray diffractometer (PANalytical, Almedo, The Netherlands). Particle size in the samples was measured by the electroacoustic spectroscopy method using a DT-1202 analyzer (Dispersion Technology Inc., New York, NY, USA) [17]. The particle size distribution of the CeO_2_-Ag samples was measured by LDA on a Shimadzu SALD-7500 nano-laser particle size analyzer (Shimadzu Corp., Kyoto, Japan).

### 2.5. Indicator Method for Determining the Concentration of Acid-Base Centers

The acid-base properties of CeO_2_ surface were analyzed using the indicator method, which involves the adsorption of indicators onto the oxide surface. This technique enables the qualitative identification and quantitative determination of active centers on the surface, as well as the distribution of active centers by their activity. Five samples were prepared for each indicator:

1. Three parallel samples (0.1 g of the analyzed oxide powder + 3 mL of the indicator).
2. Reference sample (0.1 g of the analyzed oxide powder + 2 mL of distilled water).
3. A 3 mL solution of an indicator without the sample.

The samples were mixed thoroughly and stored at 20 °C for one day. Then, before measurement, 2 mL of water was added to three parallel samples and then the pure indicator solution and the samples were mixed, and 3 mL of an indicator was added to a reference sample, after which the sample was also mixed. The resulting solutions containing a weighed portion of the test powder were centrifuged for 3× *g* min at 9000 rpm. The solutions thus prepared in this way were used for further studies on a spectrophotometer. The set of indicators used allowed the acid-base properties to be recorded in the pKa (acidity constant indicator) range from 1.3 to 12.8 [18]. Some characteristics of the indicators are shown in Table 1.

The quantitative determination of the adsorption centers (a, mmol/g) was carried out by the spectrophotometric method in the UV and visible regions of the spectrum. The investigated solutions were subjected to spectrophotometry in cuvettes 1 mm thick, relative to distilled water on a UNICO 2802 S spectrophotometer at a wavelength corresponding to the absorption maximum of each indicator (Table 1). The data obtained were used to calculate the specific adsorption. Specific adsorption (g) was calculated using the following equation:

(1) $$g=\frac{cV}{D_{0}}\cdot \left|\frac{\left|D_{0}-D_{1}\right|}{a_{1}}\pm \frac{\left|D_{0}-D_{2}\right|}{a_{2}}\right|$$

where *c* is the concentration of the indicator, mol/dm^3^, *V* is the sample volume, dm^3^; *D*_0_ is the optical density of the initial indicator, *D*_1_ is the optical density of the indicator after sorption by the sample, *D*_2_ is the optical density of the reference sample (solvent + sample), and *a*_1_ and *a*_2_ are weighed portions of the sample, *g*.

### 2.6. Determination of Photocatalytic Activity

The photocatalytic activity of the samples was studied by carrying out a model experiment based on the photodegradation of the methyl orange indicator under UV irradiation [19]. Samples were prepared as follows. An amount of 0.2 g of a sample and 0.2 mL of a 0.1% aqueous solution of methyl orange indicator were applied onto a glass substrate, evenly distributed over the surface of the substrate, and then dried for a day at room temperature. Next, the samples were placed under a UV lamp for 11 h. After each hour of exposure to UV light, the samples were photographed under identical conditions. The photographs were processed using the Adobe Photoshop CC software. The program analyzed the surface area of the sample and found its average RGB color coordinates. Next, the RGB color coordinates were converted into CIE 1931 coordinates [20]. Then, the dependences of the color coordinates of the samples in CIE 1931 space on the exposure time to UV radiation were plotted [21].

## 3. Results and Discussion

**Figure 1:**
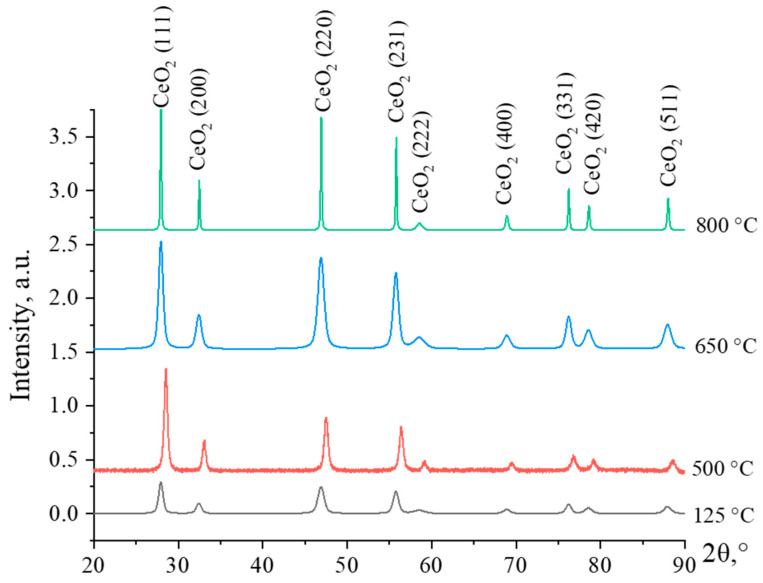
Diffraction patterns of cerium hydroxide samples, calcined at temperatures of 125, 500, 650, and 800 °C.

In an initial step, to determine the most optimal temperature for nanosized CeO_2_ synthesis, cerium hydroxide powder was calcined at temperatures of 125, 500, 650, and 800 °C. Diffraction patterns of cerium hydroxide samples calcined at different temperatures are shown in Figure 1.

The characteristics that show typical peaks of CeO_2_ with a cubic face-centered crystal lattice are presented in the diffractograms. The characteristic peaks of the sample dried at 125 °C are weakly intense and broadening, indicating that its structure is semi-amorphous. With an increase in the calcination temperature, the intensity of the characteristic peaks rises. Up to the decomposition temperature, the synthesized cerium hydroxide samples exist in the form of Ce(OH)_4_, which is an amphoteric compound. References indicate that at temperatures above 500–600 °C, complete decomposition of cerium hydroxide with the formation of CeO_2_ occurs [22].

At an annealing temperature of 650 °C, the diffraction peaks of CeO_2_ are low-intensity and broadened, which indicates a low degree of crystallinity of the material and small crystallite sizes. Increasing the calcination temperature up to 800 °C, leads to a sharp increase in the intensity of diffraction maxima, which is associated with the growth of crystallites. As a result, the intensity of particle agglomeration processes increases, and the specific surface area of the material decreases, as it was reported by Lopez and Mendoza [23]. Thus, the calcination temperature of 650 °C was chosen for the further experiments for calcining the CeO_2_-Ag samples, because at this temperature the crystal structure of the particles is fully formed, and at the same time, there is no significant agglomeration of the particles.

**Figure 2:**
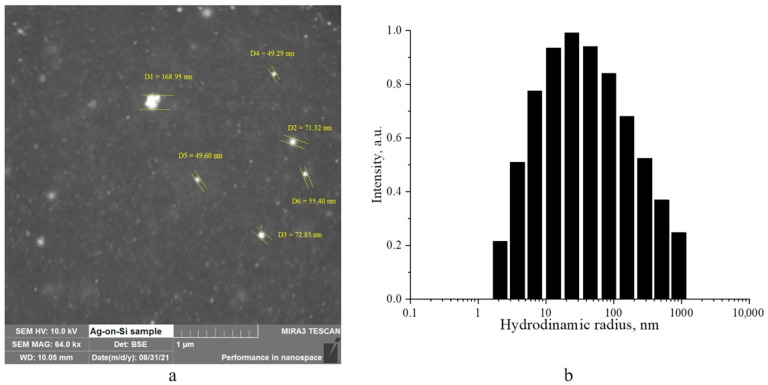
SEM micrograph of a dried sol of silver nanoparticles (**a**), and a histogram of the hydrodynamic radii distribution of silver nanoparticles (**b**).

The morphology of silver nanoparticles was studied by scanning electron microscopy (Figure 2a). Additionally, they analyzed the sol using the photon correlation spectroscopy (PCS) method to obtain information on the size distribution of the Ag nanoparticles. The results of this analysis were presented in a histogram of the hydrodynamic radii distribution, which can be seen in Figure 2b.

The presented photo clearly shows silver nanoparticles as bright white, spherical dots, thanks to the backscattered electrons (BSE) detector mode. Individual nanoparticles can reach a maximum size of ≈70 nm, while clusters of up to 10 particles are also visible. The SEM results are in good agreement with the PCS data, which show a distribution of hydrodynamic particle radii close to Gaussian, with a maximum of 20–30 nm, corresponding to an average particle diameter of 40–60 nm.

**Figure 3:**
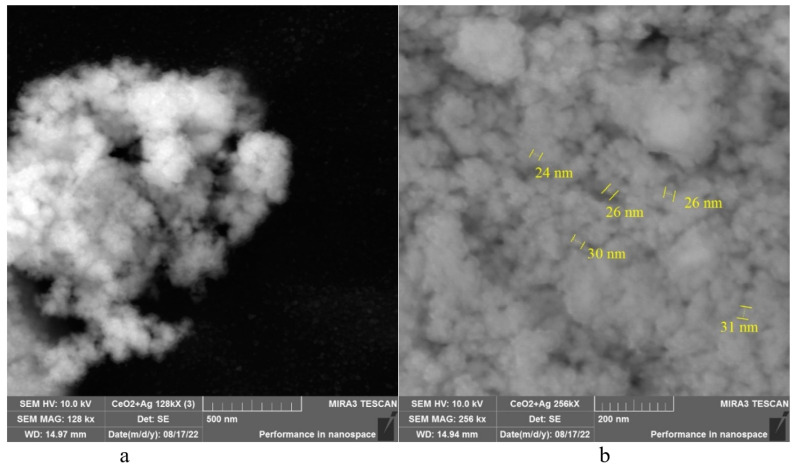
SEM micrographs of the CeO_2_-Ag4 sample: (**a**) 128,000-fold magnification; (**b**) 25,600-fold magnification.

The morphology of CeO_2_-Ag samples was studied using SEM, and all samples exhibited identical morphology, differing only in the concentration of silver nanoparticles. Figure 3 shows SEM micrographs of a CeO_2_-Ag4 sample.

CeO_2_-Ag powders are formed by particles with an average diameter of less than 30 nm. The particles are aggregated in loose agglomerates. The photographs show that the powders have a developed surface, and the particles in the powders have a largely amorphous structure and a low degree of crystallinity. To refine the granulometric composition, an electroacoustic spectroscopy study was conducted on a sample of pure CeO_2_ that had been calcined at 650 °C.

**Figure 4:**
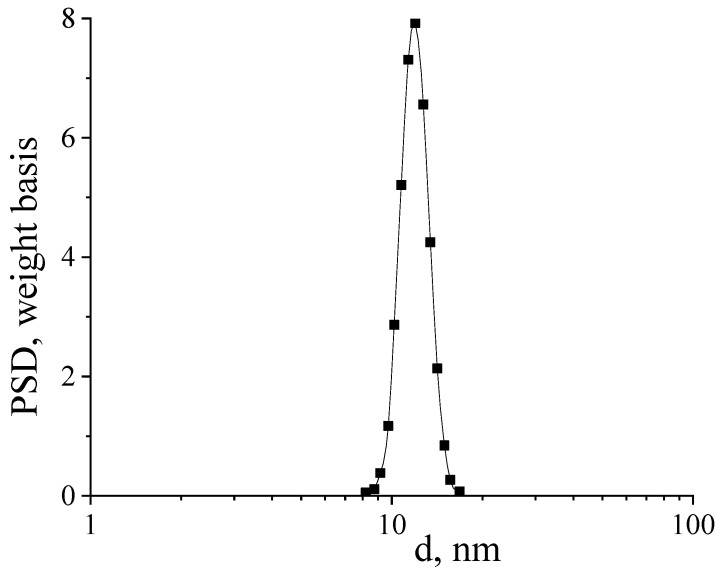
Histogram of particle size distribution in a suspension of a CeO_2_ sample redispersed in water.

Figure 4 shows a histogram of the particle size distribution of a nanosized CeO_2_ sample calcined at 650 °C.

**Figure 5:**
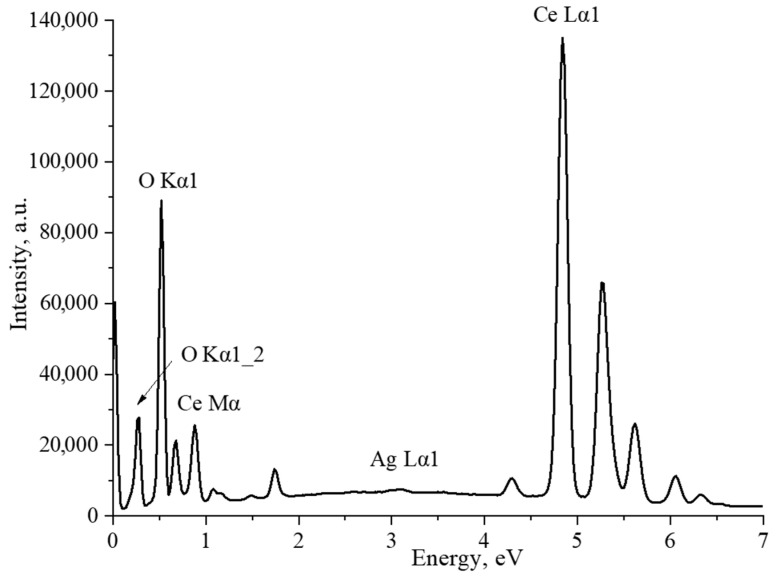
EDX spectrum of the CeO_2_-Ag4 sample.

The average particle size in the sample was 12 ± 4 nm, which is in good agreement with the SEM data. As an additional confirmation of the content of silver nanoparticles in the composites, the CeO_2_-Ag4 sample was examined by the EDX method. (Figure 5). The interpretation of the spectrum is presented in Table 2.

**Figure 6:**
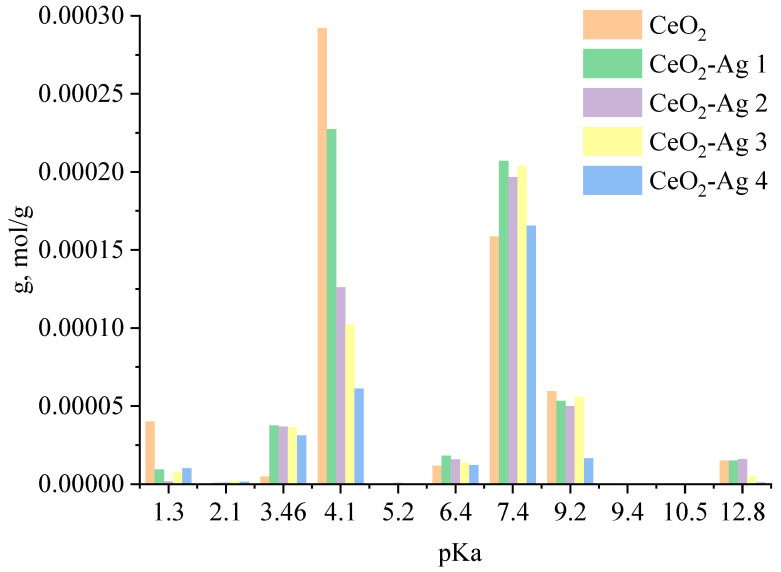
Acid-base properties of CeO_2_-Ag nanocomposites.

The spectrum displays prominent characteristic lines of cerium and oxygen. The atomic ratio of O/Ce is 1.6, which can be accounted for by the method’s higher sensitivity to cerium than oxygen due to the former’s higher atomic weight. Furthermore, a minor silver line was detected in the spectrum. According to the EDX results, the sample’s mass fraction of silver is 0.2%.In the next stage, the acid-base properties of cerium oxide nanoparticles and CeO_2_-Ag nanocomposites with different contents of silver nanoparticles were studied. The resulting dependence is shown in Figure 6.

An analysis of the dependence showed that the sample of nanosized cerium oxide contains centers with pKa = 1.3; 3.46; 4.1, 6.4; 7.4; 9.2, and 12.8. The presence of these acid-base centers is due to the existence of defects on the surface of nanosized cerium oxide: cerium vacancies (V_Ce_), oxygen vacancies (V_O_), Schottky defects, Frankel defects, etc. [24]. When a sample of CeO_2_ nanoparticles is calcined in oxygen, disordering occurs, which is accompanied by the formation of cerium and oxygen vacancies. The equation with full ionization of point defects is presented below:

(2) $${\mathrm{CeO}}_{2}\overset{t}{\to }O_{o}{+V}_{\mathrm{Ce}}^{////}{+V}_{\mathrm{Ce}}^{\cdot \cdot }+2h$$

When silver nitrate is added to the solution, silver ions interact with surface defects by the following crystal chemical equation:

(3) $${\mathrm{Ag}(\mathrm{CeO}}_{2})\to {\mathrm{Ag}}_{\mathrm{Ce}}^{///}{+2V}_{O}+3h\to {\mathrm{Ag}}_{\mathrm{Ce}}^{///}{+2V}_{O}^{\cdot }+h\to {\mathrm{Ag}}_{\mathrm{Ce}}^{///}{+2V}_{O}^{\cdot \cdot }+e$$

**Figure 7:**
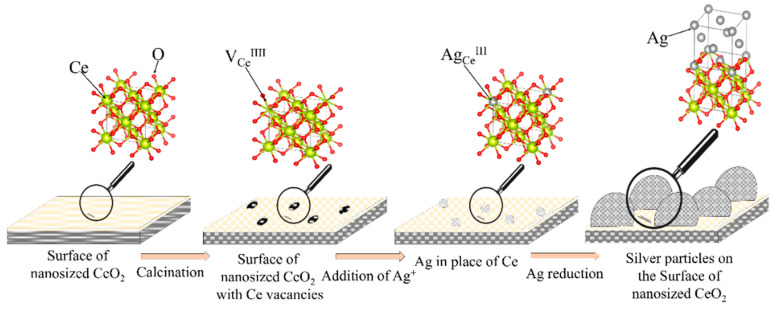
Scheme of the CeO_2_-Ag nanocomposite formation.

After the addition of sodium borohydride, the silver ions located on the surface of cerium oxide are reduced to atomic silver, which is the crystallization center of silver nanoparticles. The proposed model for the formation of the CeO_2_-Ag nanocomposite is confirmed by a decrease in the specific adsorption (g) of indicators on acid-base sites related to Bronsted acids with pKa = 4.1 (from 2.92 × 10^−4^ to 6.11 × 10^−5^) with an increase in the concentration of silver nanoparticles. Schematically, the process of formation of the CeO_2_-Ag nanocomposite is shown in Figure 7. Since the composite was obtained by calcining cerium hydroxide with silver nanoparticles at a sufficiently high temperature—650 °C, silver can exist in the composite in an oxidized form, as well as in the form of substitutional defects Ag_Ce_ [25].

Adsorption of specific functional groups, atoms, or ions on a material’s surface is influenced by active sites possessing acid or base properties. As such, the adsorption properties of a material are intricately linked to its acid-base characteristics. CeO_2_ is well known for its catalytic properties, which arise from the processes of adsorption and desorption. Silver nanoparticles have been shown to modify the acid-base and catalytic properties of cerium oxide.

In this study, we conducted a model experiment to investigate the photocatalytic properties of CeO_2_-Ag nanocomposite samples in the photodegradation of methyl orange dye under UV radiation. The surface morphology and discoloration of the samples before and after UV radiation were captured and presented in Table 3.

Our results reveal the importance of active sites and their interactions in the catalytic properties of CeO_2_, highlighting the potential of nanocomposite materials in catalysis and environmental remediation applications. The insights obtained from our study may contribute to the design of more effective and efficient heterogeneous catalysts with tailored acid-base and catalytic properties.

**Figure 8:**
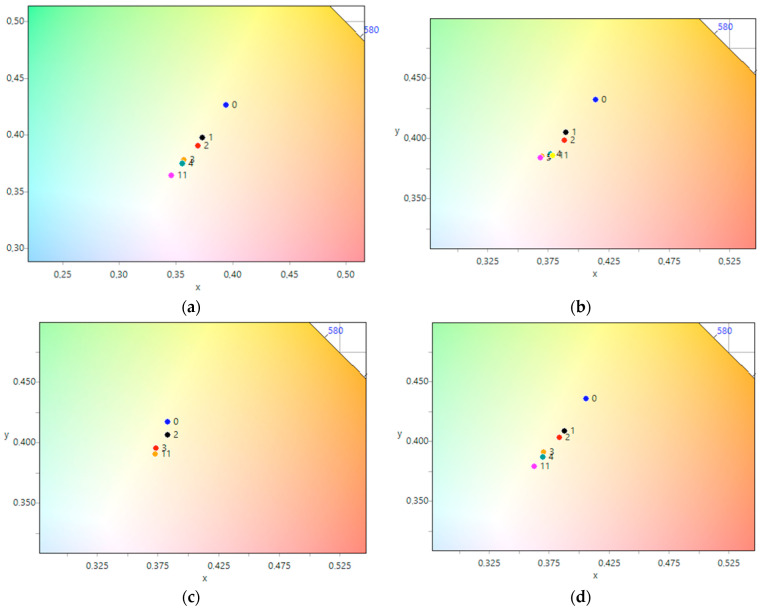

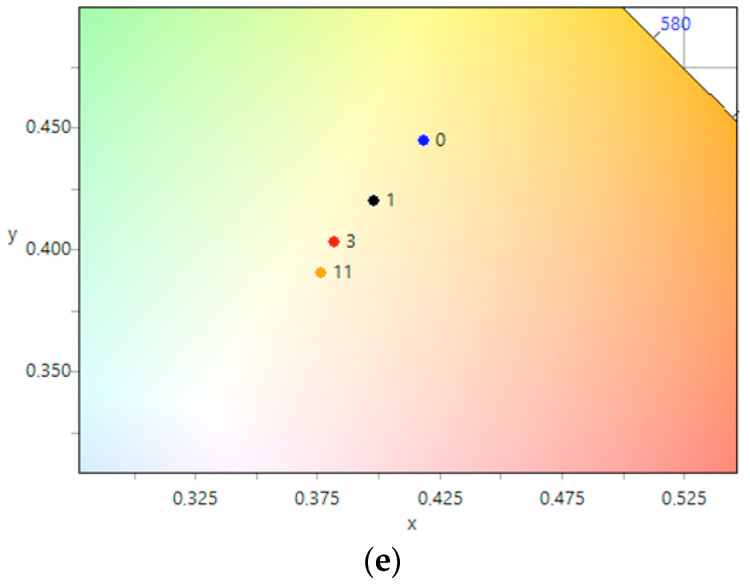
Change of color coordinates in the CIE 1931 coordinate system during the photodegradation of methyl orange: (**a**) CeO_2_, (**b**) CeO_2_-Ag1, (**c**) CeO_2_-Ag2, (**d**) CeO_2_-Ag3, (**e**) CeO_2_-Ag4.

The photographic evidence illustrates that exposure to UV light leads to a noticeable increase in surface brightness of both nanosized CeO_2_ samples, with and without silver nanoparticles. The most significant changes were observed within the first hour of exposure. To more objectively assess the color changes in each photograph, we calculated the average color tint and plotted the dependence of the average color tint on exposure time using CIE 1931 coordinates. These results are presented in Figure 8. The plotted dependencies for all five samples show a consistent trend towards shifting the color coordinate from the starting point towards the center, indicating a discoloration of the samples. As previously observed in the photographs (Table 3), the most significant shift occurred within the first hour of the experiment for all samples. It is noteworthy that for the CeO_2_-Ag1–4 samples (Figure 8b–e), the color coordinate changes were completed after 3–4 h of UV light illumination, indicating successful dye photodegradation. However, the CeO_2_ sample (Figure 8a) did not reach the final color coordinates even after 4 h, suggesting that the photodegradation process was not yet complete.

For the CeO_2_-Ag1–4 samples, no dependence of the dye photodegradation rate on the silver concentration was found. However, it can be argued that the introduction of silver into CeO_2_ enhances photocatalytic properties. These data correlate with an increase in the concentration of active sites with pKa 7.4 in all CeO_2_-Ag samples compared to pure CeO_2_.

## 4. Conclusions

In summary, the phase composition, morphology, acid-base, and photocatalytic properties of CeO_2_ and CeO_2_-Ag nanocomposites were studied. It has been found that the CeO_2_ crystalline phase is formed when calcination of cerium hydroxide powders synthesized by the sol-gel method at a temperature of 650 °C. CeO_2_ powders obtained at 650 °C are characterized by a particle size of the order of 10–20 nm and a weakly expressed crystal structure. The incorporation of silver into CeO_2_ resulted in an increase of approximately 25% in the concentration of active sites with pKa = 7.4, across all concentrations investigated in this study. Based on the experimental results, it can be asserted that nanoscale cerium oxide synthesized via annealing at 650 °C, as well as CeO_2_ nanocomposites with silver nanoparticles, exhibit significant photocatalytic activity. CeO_2_ powders annealed at 650 °C are particularly well-suited for both catalytic and phosphor doping applications due to their nanocrystalline structure and high concentration of active sites with pKa = 4.1 and pKa = 7.4, which correspond to surface defects and enhanced catalytic activity.

## Figures and Tables

**Table 1:** The main characteristics of the indicators.

No.	Indicators	pKa	λ_max_, nm
1	Brilliant green	1.3	610
2	Basic fuchsin	2.1	540
3	Methyl orange	3.46	464
4	Bromophenol blue	4.1	590
5	Methyl red	5.2	530
6	Bromocresol purple	6.4	540
7	Bromothymol blue	7.4	540
8	Neutral red	9.2	430
9	Phenolphthalein	9.4	440
10	Alizarin red	10.5	425
11	Indigo carmine	12.8	610

**Table 2:** Interpretation of the EDX spectrum of the CeO_2_-Ag4 sample.

Element	Type of Line	wt%	σ wt%	At.%
O	K-series	15.43	0.05	61.49
Ag	L-series	0.20	0.04	0.12
Ce	L-series	84.37	0.06	38.40
Summ.		100		100

**Table 3:** Photos of the surface of the samples at different times of UV radiation exposure.

Time, h	CeO_2_	CeO_2_-Ag1	CeO_2_-Ag2	CeO_2_-Ag3	CeO_2_-Ag4
0	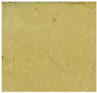	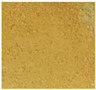	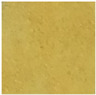	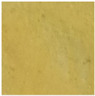	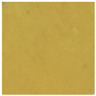
1	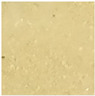	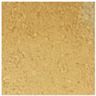	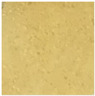	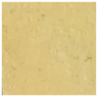	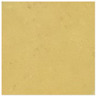
2	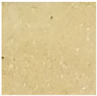	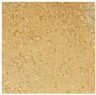	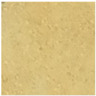	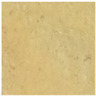	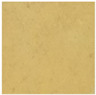
4	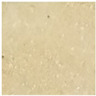	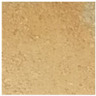	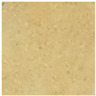	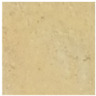	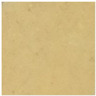
6	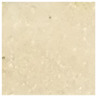	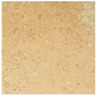	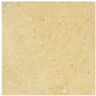	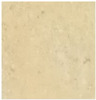	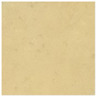
10	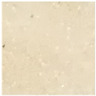	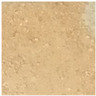	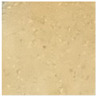	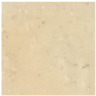	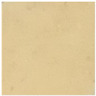

## Data Availability

All data are available upon request from the corresponding author.
